# EEG beta suppression and low gamma modulation are different elements of human upright walking

**DOI:** 10.3389/fnhum.2014.00485

**Published:** 2014-07-08

**Authors:** Martin Seeber, Reinhold Scherer, Johanna Wagner, Teodoro Solis-Escalante, Gernot R. Müller-Putz

**Affiliations:** ^1^Laboratory of Brain-Computer Interfaces, Institute for Knowledge Discovery, Graz University of TechnologyGraz, Austria; ^2^BioTechMed-GrazGraz, Austria; ^3^Rehabilitation Clinic Judendorf-Strassengel Judendorf-StrassengelAustria; ^4^Department of Biomechanical Engineering, Delft University of TechnologyDelft, Netherlands

**Keywords:** electroencephalography (EEG), gait, brain mapping, motor cortex, magnetic resonance imaging

## Abstract

Cortical involvement during upright walking is not well-studied in humans. We analyzed non-invasive electroencephalographic (EEG) recordings from able-bodied volunteers who participated in a robot-assisted gait-training experiment. To enable functional neuroimaging during walking, we applied source modeling to high-density (120 channels) EEG recordings using individual anatomy reconstructed from structural magnetic resonance imaging scans. First, we analyzed amplitude differences between the conditions, walking and upright standing. Second, we investigated amplitude modulations related to the gait phase. During active walking upper μ (10–12 Hz) and β (18–30 Hz) oscillations were suppressed [event-related desynchronization (ERD)] compared to upright standing. Significant β ERD activity was located focally in central sensorimotor areas for 9/10 subjects. Additionally, we found that low γ (24–40 Hz) amplitudes were modulated related to the gait phase. Because there is a certain frequency band overlap between sustained β ERD and gait phase related modulations in the low γ range, these two phenomena are superimposed. Thus, we observe gait phase related amplitude modulations at a certain ERD level. We conclude that sustained μ and β ERD reflect a movement related state change of cortical excitability while gait phase related modulations in the low γ represent the motion sequence timing during gait. Interestingly, the center frequencies of sustained β ERD and gait phase modulated amplitudes were identified to be different. They may therefore be caused by different neuronal rhythms, which should be taken under consideration in future studies.

## INTRODUCTION

Investigating neural dynamics during natural motor behavior is necessary to gain new knowledge about cortical involvement during motor control. This knowledge is fundamental for studying motor impairment after brain injury. We aim to develop a neurophysiological model of human gait. To address this problem, we focused on the analysis of neuronal oscillations (Buzsáki and Draguhn, 2004) gained from high-density electroencephalographic (EEG) recordings. The excellent temporal resolution of EEG recordings enables the analysis of electrocortical activity as it relates to the gait cycle phases. The spatial interpretability of the EEG can substantially be improved by applying source modeling (Baillet et al., 2001; Michel et al., 2004) to high-density EEG data. Yet, electrocortical oscillations during human upright walking are not well-studied.

Previous studies have shown that EEG spectral power in the μ and β band decreases over sensorimotor areas (Jasper and Penfield, 1949) during isolated foot movements (Pfurtscheller et al., 1997; Crone et al., 1998; Miller et al., 2007), motor preparation and motor imagery (Pfurtscheller and Neuper, 1997; Müller-Putz et al., 2007), walking on the treadmill (Severens et al., 2012) and robot-assisted walking (Wagner et al., 2012), when compared to a rest (non-movement) condition. These phenomena are classically described as event-related desynchronization and synchronization (ERD/ERS; Pfurtscheller and Aranibar, 1977; Pfurtscheller and Lopes da Silva, 1999). After the movement, β band power increases in a short-lasting burst called post-movement β synchronization (Pfurtscheller et al., 1996; Müller-Putz et al., 2007; Solis-Escalante et al., 2012). The presence of β oscillations at rest and the elevated state after movement led to the view that β oscillations represent an idling state of the motor cortex (Pfurtscheller et al., 1996). This theory has been revised with the hypothesis that β oscillations promote the maintenance of the current motor set at the expense of new movements (Engel and Fries, 2010; Jenkinson and Brown, 2011). A recent study reported significant coupling between EEG recordings over the leg motor area and electromyography (EMG) from the anterior tibialis muscle at 24–40 Hz during treadmill walking (Petersen et al., 2012). This frequency range is very similar to gait cycle modulated low γ oscillations (25–40 Hz) located in central midline areas shown from Wagner et al. (2012). Contrary to this evidence, Gwin et al. (2011) reported gait cycle phase coupled electrocortical activity in the α, β and the high γ band located in the anterior cingulate, posterior parietal, left and right sensorimotor cortex. In summary, the literature describes two major alterations of EEG oscillations during gait. First, neuronal oscillations in the μ and β band are suppressed (ERD) during movement when compared to a rest, non-movement condition. Second, their amplitudes are modulated locked to the gait cycle phase during walking.

The previously mentioned studies relied heavily on independent component analysis (ICA) and dipole analysis for spatial location identification. While this methodology has many benefits, the location of specific independent components is not necessarily consistent with the location of certain brain activity patterns, e.g., ERD/ERS. The aim of this work was to directly localize μ and β ERD activities as well as gait phase modulated oscillations. To meet this objective, we introduced a measure that quantifies the gait cycle related amplitude modulation of a certain oscillation. These activity measures were mapped on the cortex using EEG source imaging (Baillet et al., 2001; Michel et al., 2004) based on individual anatomy reconstructed from magnetic resonance imaging (MRI) scans. Furthermore, we discuss the coexistence and superposition of sustained μ and β ERD and gait phase modulated oscillations in terms of cortical location and frequency of appearance, since these phenomena have thus far only been discussed separately.

## MATERIALS AND METHODS

### EXPERIMENT

Ten healthy volunteers (S1–S10, five female, five male, 25.6 ± 3.5 years) participated in this study. The experimental procedure was approved by the ethical committee of the Medical University Graz. Each subject gave informed consent before the experiment. Participants completed 4 runs (6 min each) of active walking and 3 runs of upright standing (3 min each) in a robotic gait orthosis (Lokomat, Hocoma, Switzerland). Walking speed was constant and adjusted to the participants’ leg length ranging from 1.8 to 2.2 km per hour. Body weight support was adjusted with the help of experienced physical therapists to less than 30% in every participant. The Lokomat was operated with 100% guidance force. This set-up was chosen to ensure a well-controlled and steady gait pattern during the experiment. Participants were trained to walk in a natural way in the Lokomat and were asked look straight ahead and to blink normally to avoid eye artifacts during the experiment.

### RECORDINGS

120 EEG channels were recorded by combining four 32-channel amplifiers (BrainAmp, Brainproducts, Munich, Germany). To determine the electrode positions and anatomical landmarks (nasion, vertex, left- and right pre-auricular points) for each subject, we used a 3D localizer (Zebris Elpos system, USA). Structural T1 MRI scans were recorded in a post-screening session using a 3.0 Tesla (Tim Trio/Skyra, Siemens, Erlangen, Germany) scanner. EEG was sampled to 2.5 kHz, high pass filtered at 0.1 Hz and low pass filtered at 1 kHz. The electrode montage was in accordance with the 5% international 10/20 EEG system (EasyCap, Germany; Oostenveld and Praamstra, 2001). Reference and ground electrodes were placed on the left and right mastoids respectively. Electrode impedances were <10 kΩ. Foot contact was measured by electro-mechanical switches placed over the calcaneus bone at the heel of both feet. We defined one gait cycle as the interval between two right leg heel contacts. More detailed information about the experimental set-up and procedure can be found in Wagner et al. (2012).

### DATA ANALYSIS

#### Preprocessing and artifact correction

The EEG recordings were high pass filtered at 1 Hz [zerophase FIR filter order 7500] and low pass filtered at 200 Hz [zerophase FIR filter order 36]. To reduce computation time and required memory for time-frequency (TF) analysis, the data was down sampled to 250 Hz. EEG data from the active walking (gait) condition was epoched and time warped according to the mean gait cycle duration for every subject individually (group mean 2.13 ± 0.17 s). EEG data from the upright standing (rest) condition was sliced into non-overlapping segments with the length of the mean gait cycle duration. An EEG channel was not used if its variance was >2 times the median variance of all channels or if its kurtosis was >5 resulting in 99.28 ± 7.36 retained channels. An entire trial was rejected if more than half of the electrodes were excluded based on the previously mentioned criteria. Finally, an average of 219.3 (range 89–484) gait trials and 231.6 (range 130–301) rest segments were used for analysis. EEG was re-referenced according to the common average and the trials were corrected for direct current (DC) offsets.

#### EEG source modeling

To enable neuroimaging during the gait paradigm, we applied inverse mapping to high-density EEG recordings using a distributed source model based on individual anatomy. Source imaging of high-density EEG data (Baillet et al., 2001; Darvas et al., 2004; Michel et al., 2004) is increasingly evolving into a capable brain imaging method (Michel and Murray, 2012) due to advances in signal processing and the availability of computational power. The capability of high-density EEG source imaging based on sophisticated head models using individual anatomy has been demonstrated by Brodbeck et al. (2011) in a large-scale clinical study. Thus, we computed realistic head models as boundary element model (BEM) consisting of four surface layers (brain, inner skull, outer skull, head surface) that were reconstructed from individual structural T1 MRI scans. Cortical reconstruction and volumetric segmentation was performed with the Freesurfer image analysis suite (Dale et al., 1999; Fischl, 2012)^1^. The BEM model and EEG electrode positions were co-registered using four anatomical landmarks (nasion, vertex, left- and right pre-auricular points). The bioelectric forward problem was formulated as distributed source model using OpenMEEG (Kybic et al., 2005; Gramfort et al., 2010), in which 15000 sources were oriented perpendicular to the reconstructed gray matter cortical surface. This number of sources was necessary to model the folded brain surface and to take into account the individual gyri and sulci of each individual. To solve the ill-posed inverse problem, we used the sLORETA approach (Pascual-Marqui, 2002). The noise covariance matrix was calculated from the rest EEG segments and was used for whitening the lead field matrix. These analyses were performed with the Brainstorm toolbox (Tadel et al., 2011)^2^.

#### Time-frequency decomposition of EEG sources

To enable the computation of functional topographies that can describe the dynamics of different brain rhythms during the gait cycle, we analyzed the EEG sources in the TF domain preserving the high temporal resolution of the EEG. Based on previously demonstrated utility in EEG analysis (Tallon-Baudry and Bertrand, 1999), we used Morlet et al. (1982) wavelets for TF decomposition. To design the mother wavelet, we set the full width half maximum value to 3 s for the Gaussian kernel at a center frequency of 1 Hz. Because TF decomposition is linear, we performed it in the sensor space before applying the linear inverse method to reduce computational cost. TF magnitudes were then calculated in the source space. This analysis results in brain topographies for each center frequency (4–50 Hz, 2 Hz steps) and every time sample. To enhance the signal to noise ratio (SNR) of the topographies, we averaged the TF magnitudes for the gait and rest condition segments respectively.

#### μ and β ERD source imaging

Logarithmic (natural logarithm) amplitude ratios of the gait and rest condition were computed in the EEG source space. These topographies illustrated relative amplitude changes between the gait and rest condition for a specific frequency. We applied a logarithmic function to give an activity scale that was centered at 0 and avoid the strong effect effect of outliers on power spectrum values induced by squaring. In noisy data squaring has the effect to amplify outliers relative to the signal of interest. Although logarithmic amplitude ratio is distinct from the classically defined ERD/ERS, it describes the same phenomena namely a relative spectral change between a defined task and reference period, in our case walking relative to upright standing. Because the terms ERD/ERS are well established in EEG analysis community, we continue using these terms.

To show general, sustained ERD/ERS activity over the whole gait cycle we calculated temporal mean ERD/ERS topographies for every frequency. The individual μ (8–13 Hz) and β (13–30 Hz) center frequencies were identified from local extreme ERD values for these bands. These ERD extremes were obtained from a global, spatially independent spectrum calculated from the population mean of the 150 source vertices (1% of the source space vertices) in the whole source space that showed most ERD. We used this approach to assure the identification of sensorimotor rhythms (μ and β) was fully data driven, without any *a priori* spatial region of interest (ROI). For the resulting μ and β center frequencies, we obtained ERD/ERS topographies, which showed significant ERD in the sensorimotor cortex. We clustered these significant vertices in sensorimotor cortex to identify an individual brain ROI in every subject. This cluster was reduced to the 150 sources (1% of the source space vertices) where ERD was greatest to obtain the same ROI size for each subject.

#### Gait phase modulated oscillatory amplitudes

To analyze the modulation of oscillatory amplitudes relative to the gait cycle, we calculated logarithmic magnitude ratios for each time point relative to the mean TF magnitude of the whole gait cycle. These activities therefore express relative spectral changes across the gait cycle, not changes relative to the rest condition. To identify and localize the EEG frequency components with amplitude modulations most strongly locked to the gait cycle, we introduce the gait phase modulation (GPM) measure. To do so, we calculated a modified version of the modulation index that has previously been used to quantify the phase amplitude coupling (Canolty et al., 2006) of slow to fast neuronal oscillations. Here, we generated a phase signal according to the gait cycle, assuming a two periods pattern.

$$GPM\left(f\right)=\frac{2}{\sqrt{2}\cdot \sigma_{A\left(f\right)}}\cdot \overset{N-1}{\underset{n=0}{\sum }}A\left(n,f\right)\cdot e^{-2\pi i\cdot \frac{2\cdot n}{N}}$$

**FIGURE 1:**
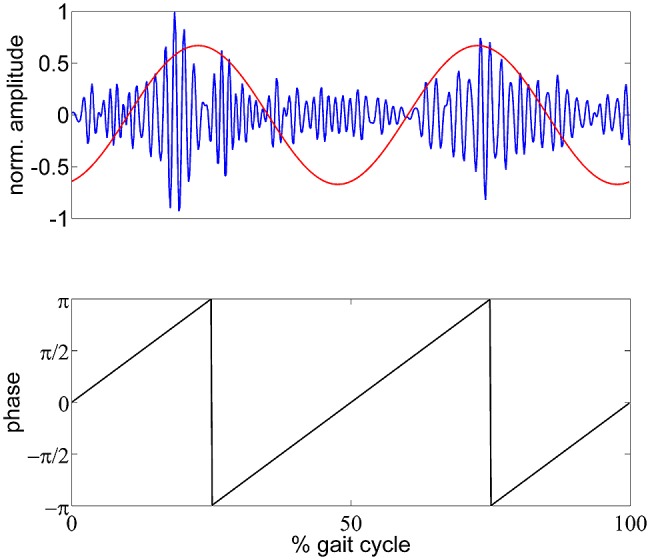
**Schematic illustration of the GPM measure.** Top panel: exemplary (single trial) gait phase modulated low γ oscillation in blue and reconstructed GPM as time course. Bottom panel: generated gait phase assuming a two period pattern per gait cycle.

In this formula, A denotes the TF magnitude at a certain frequency f and sample point n. N is the total number of samples per gait cycle. σ _A(f)_ is the standard deviation of A and was multiplied with $\frac{2}{\sqrt{2}}$ to normalize the modulation measure. The GPM is a complex number which has a magnitude of 1 if A is modulated sinusoidally with the step frequency. The angle of A expresses the reconstructed phase of the modulation. This formulation is equivalent to the scaled discrete Fourier transform (DFT) component of the TF magnitude at the step frequency, and therefore can be back-transformed using the inverse DFT. This would result in a representation as a sinusoid with the step frequency having the magnitude and phase of the GPM. **Figure 1** shows an exemplary low γ amplitude modulation and the reconstructed GPM. This measure was calculated for multiple frequencies (4–50 Hz, 2 Hz steps) and every vertex in the source space. The resulting functional topographies show the actual cortical origin of the GPM. Additionally, we can evaluate whether the GPM are caused by EMG activities, since EMG artifacts occur as superposition in EEG recordings. Considering electrical volume conduction it is likely that EMG sources get mapped to regions close to the muscle locations, especially at sites near the neck muscles. If the GPM were caused by EMG activities these modulations would be larger in brain regions near the muscles than in central sensorimotor cortical areas.

#### Statistical non-parametric mapping (SnPM)

In statistical analysis of functional brain topographies, it is important to control for Type I errors due to the multiple comparison problem inherent to a large number of vertices or sources in a model. The family-wise error rate (FWER) was controlled by applying SnPM using permutation tests (Nichols and Holmes, 2001; Maris and Oostenveld, 2007). We pooled the single trial topographies from the gait and rest condition. From this pooled data two random subsets were drawn with permuted labels from the gait and rest condition. The logarithmic ratio of the two random subset means was computed, resulting in random ERD/ERS topographies calculated from the actual data with permuted condition labels. This procedure was performed for 10^4^ permutations. Activity was indicated as significant if its value was larger than the 95% of the maximum activity values from 10^4^ random topographies, resulting in FWER < 0.05. To evaluate GPM chance levels, we destroyed the temporal order between the trials. To this end, we shifted the TF magnitudes in time using randomly (uniform distribution) drawn time lags between 0 and the mean gait cycle period. We calculated the mean over these temporally shifted trials, resulting in topographies. Again, we performed 10^4^ permutations and set thresholds of 5% for the topographies. We ranked clustered activity according to the cluster size. Activity was deemed significant if a cluster was larger than 95% of the largest clusters from 10^4^ permutations.

To evaluate the chance level of the GPM in the central sensorimotor ROI for different rhythms we performed the same time shift procedure as described above. Here we calculated the mean GPM in the ROI, ranked the randomly observed GPM values, and deemed the GPM significant if its magnitude was greater than 95% of the values from 10^4^ permutations.

## RESULTS

### μ AND β ERD SOURCE IMAGES

**FIGURE 2:**
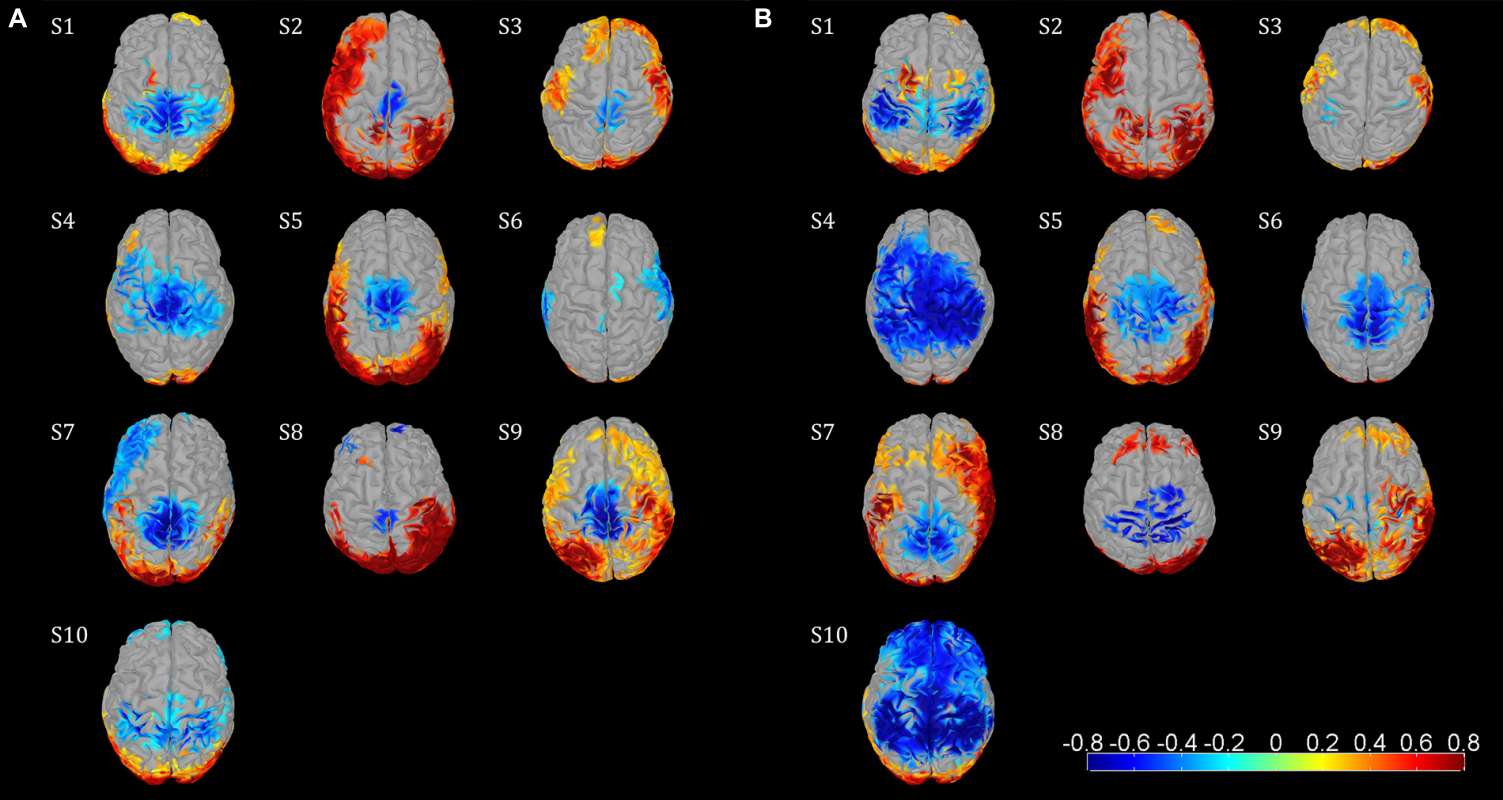
**Functional topographies of the 10 subjects illustrating significant μ and β ERD in the sensorimotor cortex during the active walking compared to the upright standing. (A)** β rhythm topographies, activity in log spectral magnitude ratios. Significant (non-parametric permutation tests, corrected *p* < 0.05) spectral decrease (ERD) is illustrated in blue, spectral increase in red. **(B)** μ rhythm topographies, figure setting as in **(A)**. Individual center frequencies were used for the μ and β rhythm in every subject which showed most ERD, whereas all μ peaks were identified at 10–12 Hz and all β peaks were between 18–30 Hz; the specific frequencies are listed in **Table 1**. Based on these functional topographies the central sensorimotor ROI was identified individually for every subject.

Event-related desynchronization and synchronization topographies showed significant ERD in sensorimotor areas for the μ and β rhythm. Significant β ERD was visible focused in central sensorimotor areas (**Figure 2A**) in 8/10 Subjects. S6 showed weak, but still significant activity in central sensorimotor areas, while S10 showed a different pattern in the sensorimotor area. We also observed significant μ ERD for most of the subjects (**Figure 2B**), which were spatially less consistent across subjects than the β activity patterns. S6 and S8 showed fewer than 150 sources with significant activity in the sensorimotor cortex for the β topography. So, we used a smaller cluster of 69 sources for S8. The focal μ cluster in the central sensorimotor areas was downsized to a size of 150 sources and used as ROI for S6. Activities in **Figure 2** illustrated in red are likely to be caused by EMG artifacts and not by ERS brain activities.

### GAIT PHASE MODULATED AMPLITUDES

**FIGURE 3:**
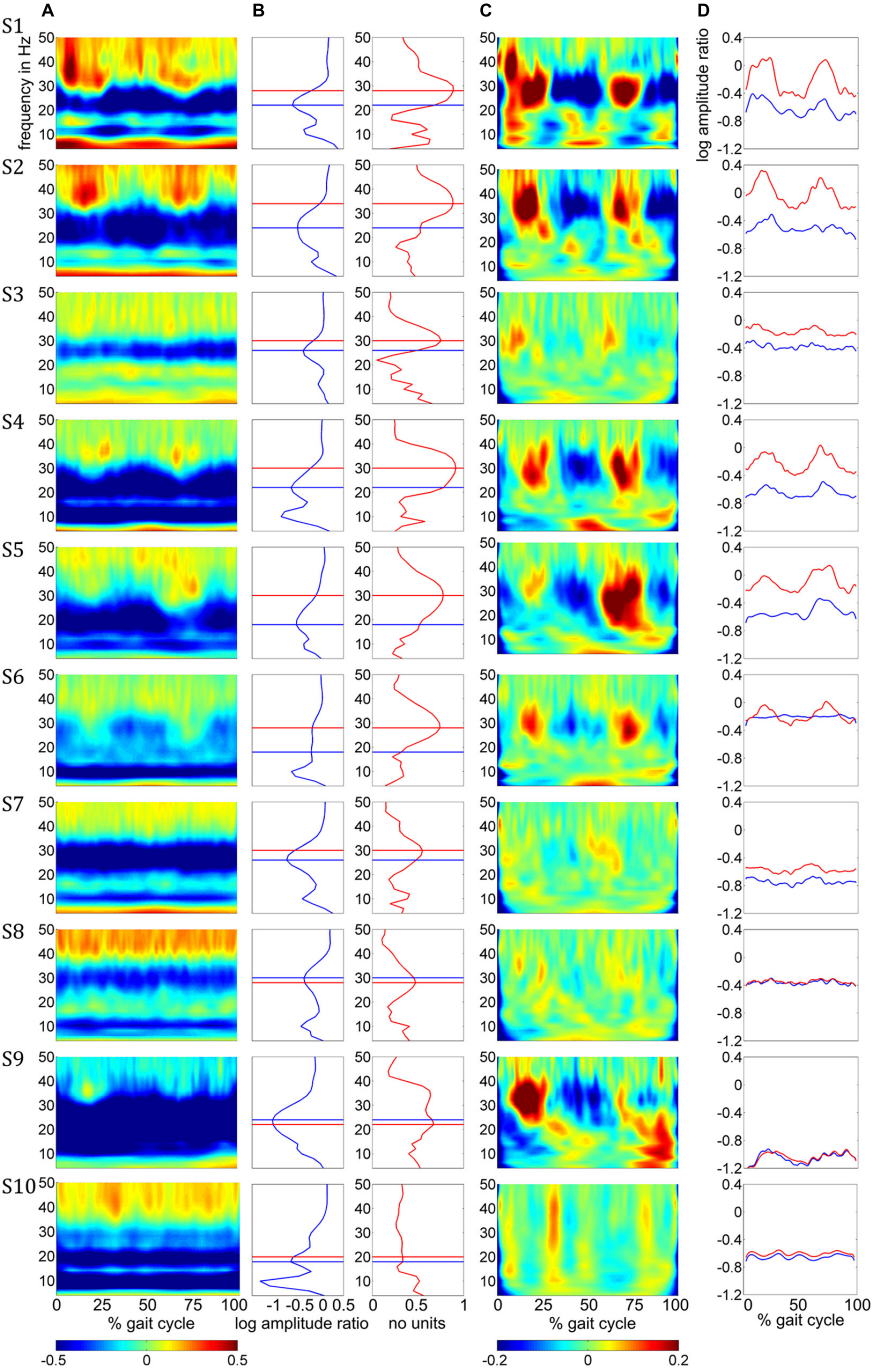
**(A)** Time-frequency ERD/ERS plots from the 10 subjects illustrating spectral changes between active walking and upright standing. Activity in log spectral ratios, spectral de/increase in blue/red **(B)** ERD (blue) and GPM (red) as a function of frequency. **(C)** TF plots illustrating amplitude modulations relative to the gait cycle mean activities. **(D)** Time courses for the β rhythm (blue) and GPM (red) center frequency. The negative offsets in these plots represent sustained ERD while the temporal modulation indicates the GPM. All plots in this figure show mean activity in the individual central sensorimotor ROI for each subject.

Amplitude changes were found in central sensorimotor ROI, both from walking to standing and across the gait cycle (**Figure 3**). ERD/ERS activities during the gait cycle show sustained μ and β ERD during the entire gait cycle (**Figure 3A**). There were pronounced amplitude modulations relative to the mean gait cycle activity at 25–40 Hz with similar temporal dynamics locked to the gait cycle in 9/10 Subjects (**Figure 3C**). Subject 10, who already showed unusual μ and β brain topographies, also featured different TF patterns. A comparison of the temporal dynamics of TF magnitudes at μ and β frequencies compared to GPM center frequencies revealed that the GPM magnitudes were significantly higher (Wilcoxon signed rank) at the GPM peak frequency than at the μ (*p* = 0.0049) and the β (*p* = 0.002) center frequencies (**Table 1**; **Figure 3D**). While sustained ERD is by definition maximal at β center frequencies, the temporal amplitude modulation is bigger at the peak GPM frequencies (**Figures 3A,D**). Moreover, the GPM frequencies were significantly different (Wilcoxon signed rank) from the μ (*p* = 0.002) and β (*p* = 0.0137) center frequencies (**Figure 3B**). This indicates that sustained μ and β ERD and GPM occur in different neuronal rhythms. The GPM activities were localized to the central sensorimotor areas for 8/10 Subjects.

**Table 1:** μ, β rhythm and modulation center frequencies (f) in Hz with the corresponding GPM values.

	f_μ_	f_β_	f_mod_	GPM_μ_	GPM_β_	GPM_mod_	P_mod_
S1	12	22	28	0.603	0.679	0.887	0.0001
S2	10	24	34	0.384	0.522	0.884	0.0001
S3	12	26	30	0.440	0.492	0.753	0.0001
S4	10	22	30	0.312	0.781	0.914	0.0001
S5	10	18	30	0.355	0.515	0.777	0.0001
S6	10	18	28	0.333	0.318	0.741	0.0001
S7	10	26	30	0.386	0.478	0.549	0.0001
S8	10	30	28	0.408	0.456	0.475	0.0015
S9	12	24	22	0.475	0.657	0.673	0.0001
S10	10	18	20	0.517	0.334	0.333	0.0120
Median	10	23	29	0.397	0.503	0.747	0.0001

## DISCUSSION

We directly localized μ and β ERD and gait phase modulated amplitudes during upright walking in humans using EEG source imaging. To investigate and quantify the cortical origin and frequency spectrum of gait phase modulated oscillations, we introduced the GPM measure.

Upper μ (10–12 Hz) and β (18–30 Hz) rhythms were suppressed (ERD) during the whole gait cycle, while low γ (25–40 Hz) oscillations were dynamically modulated related to the gait cycle phase. β ERD and low γ GPM were both localized in central sensorimotor areas. Interestingly, ERD and GPM center frequencies were identified to be different. They may therefore be caused by different neuronal rhythms.

### μ AND β ERD SOURCE IMAGES

The ERD/ERS brain topographies showed β ERD patterns in central sensorimotor areas. These spatial patterns are consistent with the classical somatotopic location of lower extremities in the human motor cortex (Jasper and Penfield, 1949). Furthermore, these patterns coincide with results for invasive electrocorticographic (ECoG) recording studies that showed β spectral power decreased in central sensorimotor areas during isolated leg movements (Crone et al., 1998; Miller et al., 2007). The variability of functional somatotopy across individuals has been reported before (Crone et al., 1998; Miller et al., 2007). Yet, the inter-subject consistent β ERD pattern located in central sensorimotor areas was a robust feature of our study. ERD/ERS topographies illustrate general, conditional spectral changes between active walking and upright standing, since there was no temporal information considered in this measure. ERD in the μ and β rhythm consequently describe general state changes for these oscillations during walking relative to a non-movement condition. ERD was interpreted as an electrophysiological correlate of activated cortical areas that are involved in sensory or cognitive processing, or in the production of motor behavior (Pfurtscheller and Lopes da Silva, 1999). Following this theory the sustained ERD reflects an active state of the sensorimotor areas during walking.

### SUPERPOSITION OF SUSTAINED ERD AND GAIT PHASE MODULATED AMPLITUDES

**FIGURE 4:**
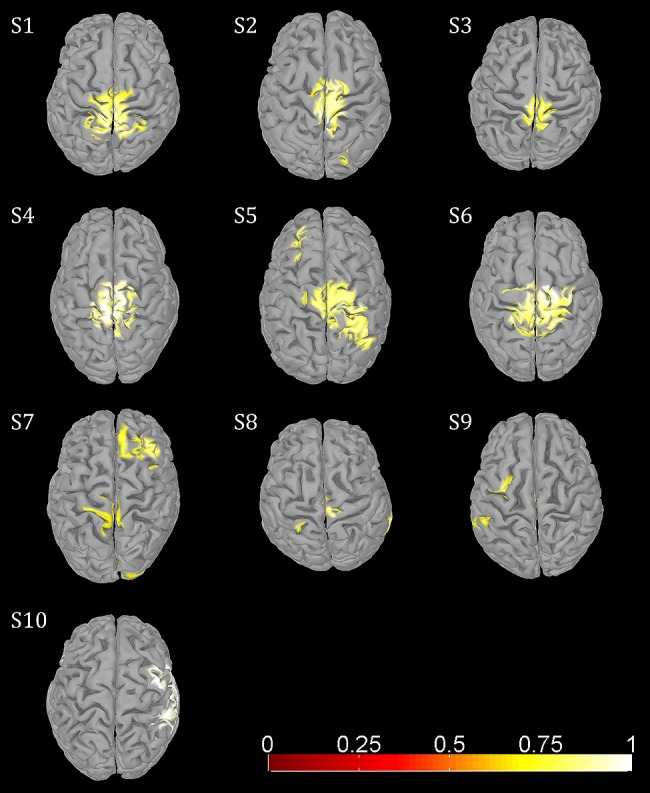
**Functional topographies of significant (non-parametric permutation tests, corrected FWER <0.05) gait phase modulated oscillations (GPM) at individual GPM center frequencies (Table 1)**.

In addition to sustained μ and β ERD, during the whole gait cycle, oscillatory amplitudes are modulated relative to the gait phase in the low γ band. These two phenomena occur simultaneously during walking and are superimposed, both in spatial location and frequency range. The GPM values are significantly larger in the low γ than at μ and β center frequencies (**Figure 3**, **Table 1**). This finding shows that GPM values, which are largest for frequencies between 28 and 36 Hz, cannot be explained exclusively by modulations of the μ and β rhythm. These modulations in the low γ are strongly linked to the gait cycle phase (**Figure 3C**, **Table 1**). The GPM maxima are clustered and located isolated in central sensorimotor areas (**Figure 4**). This localization suggests that the GPM are caused by brain signals and not by EMG activities, considering the location of head muscles and electrical volume conduction. Moreover, large GPM is present in a limited frequency band (24–40 Hz), not as broadband activity, which was reported to be associated with motion or muscular artifacts during walking (Castermans et al., 2014). The gait phase related rhythm in the low γ band was previously reported by our group (Wagner et al., 2012) using ICA and dipole reconstruction for localization. Again, the location of particular independent components is not necessarily congruent with the location of ERD/ERS activity or the GPM measures we introduced in the present work. Here, we directly localized μ and β ERD and low γ GPM using inverse modeling in a distributed source model. Moreover, our results show that μ and β ERD occur at different frequencies than the GPM, which are driven by a different rhythm in the low γ range.

The superposition of μ and β ERD and GPM is shown in **Figure 3**, where the overlay of these two phenomena can be determined to be dependent upon the frequency overlap (**Figure 3B**, **Table 1**) of the β ERD and the GPM center frequencies. In some subjects, the β ERD “covers” the GPM in these plots. The isolated gait phase related modulations are illustrated in **Figure 3C**. Because gait cycle mean activities were used as a reference, sustained ERD/ERS activities disappear in these plots. The time courses in **Figure 3D** additionally outline the coexistence of sustained ERD and GPM. Depending on the difference of the β and GPM center frequencies, these time courses differs in terms of their offset (ERD/ERS) or their temporal modulation (related to GPM).

### POTENTIAL LIMITATIONS OF THE CURRENT STUDY AND FUTURE WORK

We investigated robot-assisted walking in the Lokomat for several reasons. First, the robotic gait orthosis ensures a very steady gait pattern during the experiment. Second, the participants’ trunk can be stabilized to some extent using body weight support, which reduces movement artifacts. Third, we plan to study electrophysiology during stroke rehabilitation in future studies using the Lokomat. Nonetheless, Lokomat walking is similar but still different from treadmill walking (Hidler and Wall, 2005; Hidler et al., 2008). The generalizability of our findings therefore has to be investigated for everyday walking tasks in the future. However, our findings may be helpful both for analysis of EEG data in more naturalistic human walking tasks on one hand as well as ultimately for clinical research on the other hand.

Lower extremities are both represented close to the midline in the human motor cortex (Jasper and Penfield, 1949). Due to this cortical location and the low spatial resolution of the EEG, we are limited to reporting summed activity from both feet. In this study, it is not possible to discuss EEG activity in relation to the movement of a particular foot. For example, increased amplitudes during 10–30% of the gait cycle could be related ether to the initial and midswing phases (10–37%) of the left foot or to the mid stance phase (10–30%) of the right foot. We are therefore careful in discussing TF activity in relation to certain phases of a particular foot.

### FUNCTIONAL MEANING OF β ERD AND LOW γ MODULATION

In this section, we discuss our findings in relation to previous studies in terms of frequency ranges of the gait modulated oscillations and their cortical origin. The frequency ranges we report in this study for sustained β ERD (18–30 Hz) and the GPM (24–40 Hz) are very similar to corticomuscular coherence (CMC) peak frequencies from a recent study. In Petersen et al. (2012), the synchrony of EEG and EMG signals were investigated during treadmill walking. The authors reported CMC peaks located at the vertex (at the Cz electrode) for frequencies at 15–30 Hz for static contraction of the anterior tibial muscle, but for frequencies of 24–40 Hz for slow and normal walking. The drift of CMC peak frequencies from the β range during isometric movement tasks toward the low γ range during phasic movements has been previously discussed for upper limbs (Marsden et al., 2000; Omlor et al., 2007) and lower limbs (Gwin and Ferris, 2012). Considering these findings and assuming that different neuronal oscillations reflect different functional networks (Buzsáki and Draguhn, 2004; Schnitzler and Gross, 2005), the CMC peak drift from β frequencies during isometric movement toward low γ frequencies during phasic movements could be explained by different cerebral networks associated with the β rhythm and the low γ we identified and distinguished in this work.

The presence of β oscillations is related to maintenance of the current motor set (Engel and Fries, 2010) and promotes tonic activity at the expense of voluntary movement (Gilbertson et al., 2005; Brown, 2007; Pogosyan et al., 2009; Jenkinson and Brown, 2011). Thus, β ERD may reflect the suppression of an inhibitory network and signifies a neuronal state during walking that enables voluntary movement. We argue that β ERD during walking reflects a sustained active, movement related neuronal state, which is present during the whole gait cycle.

The most recent work from our group investigated different interactive virtual environment (VE) tasks during robot-assisted walking (Wagner et al., 2014). The authors reported low γ (23–40 Hz) gait cycle related modulations in the premotor cortex to be dependent on the VE task. This dependence was greatest during 10–30 and 60–80% of the gait cycle and was suggested to represent processes involved in motor planning. In local field potential (LFP) recordings, low γ frequencies at 25–40 Hz have been shown to modulate the firing rate of macaque primary motor neurons during a center-out brain-machine interface task (Canolty et al., 2010). γ synchronization is a fundamental process in cortical computation (Fries et al., 2007; Fries, 2009) and facilitates the coordination of distributed functional cell assemblies (Canolty et al., 2010). Consequently, the gait phase modulated low γ oscillations we report could be involved in gait phase dependent local synchronization of neuronal populations linked to sensorimotor processing or integration. The particular involvement of low γ oscillations in motor control will be investigated in future work.

μ and β ERD and gait phase related amplitude modulations are simultaneously present during walking. Following the view that neuronal oscillations at different frequencies are involved in different cortical networks (Buzsáki and Draguhn, 2004; Schnitzler and Gross, 2005; Siegel et al., 2012), our findings suggest that the sustained μ and β ERD reflect altered states of the associated networks during walking. Furthermore, another network related to the low γ rhythm may be modulated dynamically locked to the gait cycle phase. Gait phase related low γ amplitude modulation and sustained μ and β suppression may therefore be organized in different neuronal networks.

## Conflict of Interest Statement

The authors declare that the research was conducted in the absence of any commercial or financial relationships that could be construed as a potential conflict of interest.

## Footnotes

1 http://surfer.nmr.mgh.harvard.edu/

2 http://neuroimage.usc.edu/brainstorm
