# Corrigendum: EEG beta suppression and low gamma modulation are different elements of human upright walking

**DOI:** 10.3389/fnhum.2015.00542

**Published:** 2015-10-02

**Authors:** Martin Seeber, Reinhold Scherer, Johanna Wagner, Teodoro Solis-Escalante, Gernot R. Müller-Putz

**Affiliations:** ^1^Laboratory of Brain–Computer Interfaces, Institute for Knowledge Discovery, Graz University of TechnologyGraz, Austria; ^2^BioTechMed-GrazGraz, Austria; ^3^Rehabilitation Clinic Judendorf-StrassengelJudendorf-Strassengel, Austria; ^4^Department of Biomechanical Engineering, Delft University of TechnologyDelft, Netherlands

**Keywords:** electroencephalography (EEG), gait, brain mapping, motor cortex, magnetic resonance imaging

In the Original Research Article there is a missing normalization by “*N*” in the formula on page 3. This formula describes the gait phase modulation (GPM) measure. The corrected formula is written below.

$$GPM(f)=\;\frac{2}{\sqrt{2}\;\cdot \;\sigma_{A(f)}\;\cdot \;N}\;\cdot \;\sum_{n=0}^{N-1}A\left(n,f\right)\;\cdot \;e^{-2\pi i\cdot \frac{2\cdot n}{N}}$$

In our original article, the description of the GPM formula as well as the reported results are correct. As properly stated in the commentary from Trenado (2015) to our original article, the normalization by *N* is necessary to scale the *GPM* magnitude in an interval from 0 to 1. In our calculations this normalization was already applied what is represented in the GPM values we reported in Table 1 and Figure 4 in the original publication.

In contrast to Trenado (2015) we suggest to use the GPM measure as complex number, not only it's magnitude. The GPM magnitude expresses it's strength, while the GPM angle represents the phase lag between behavior and amplitude envelop at a given carrier frequency and location in the brain. In our opinion it is a benefit of the GPM measure not only to describe the correlation between amplitude envelops of brain oscillations and behavior, i.e., walking patterns, but also to provide their phase relation.

## Conflict of interest statement

The authors declare that the research was conducted in the absence of any commercial or financial relationships that could be construed as a potential conflict of interest.
